# Erkenntnisse aus 10 Jahren CIRS‑AINS: Eine Analyse von Nutzerverhalten und Ausblick auf neue Herausforderungen

**DOI:** 10.1007/s00101-020-00829-z

**Published:** 2020-08-17

**Authors:** C. Neuhaus, M. Holzschuh, C. Lichtenstern, M. St.Pierre

**Affiliations:** 1 Klinik für Anästhesiologie, Universitätsklinikum Heidelberg, Im Neuenheimer Feld 110, 69120 Heidelberg, Deutschland; 2 Anästhesiologische Klinik, Universitätsklinikum Erlangen, Erlangen, Deutschland; 3 Anonymisierungs- und Analyseteam CIRS-AINS, BDA/DGAI, Nürnberg, Deutschland

**Keywords:** Zwischenfallmanagement, Meldesysteme, Zwischenfallprävention, Incident Reporting, Patientensicherheit, Safety management, Reporting systems, Accident prevention, Critical incident reporting, Patient safety

## Abstract

**Hintergrund:**

„Critical-incident-reporting“-Systeme (CIRS) dienen dazu, Organisationen und Individuen für bislang unbekannte und sicherheitsrelevante Ereignisse zu sensibilisieren und dadurch Veränderungen herbeiführen zu können. In den letzten Jahren häufen sich allerdings kritische Stimmen, die Einsatz und Nutzen von CIRS in der Medizin hinterfragen, u. a. aufgrund unklarer bzw. zu allgemeiner inhaltlicher Kriterien für die Aufnahme einer Meldung in ein System. Ziel der Arbeit ist die Auswertung und Analyse aller Fälle aus CIRSmedical Anästhesiologie (CIRS-AINS) als Grundlage weiterer, differenzierter Betrachtungen.

**Methode:**

In einer retrospektiven Datenbankanalyse wurden alle Fälle aus CIRS-AINS (April 2010–Juni 2019) inhaltlich analysiert.

**Ergebnisse:**

Die gemeldeten 6013 Fälle setzen sich aus 3492 „incidents“ (58,1 %), 1734 „near-misses“ (28,8 %) und 787 sonstigen Meldungen (13,1 %) zusammen. Unter Letztere fielen 21 Konfliktmeldungen (0,4 %), 102 Beschwerden (1,7 %), 89 Überlastungsanzeigen (1,5 %) sowie 575 sonstige Meldungen, die keine CIRS-Fälle darstellen (9,6 %). Seit 2015 zeigt sich eine stetige Zunahme der sonstigen Meldungen um das 2,8-Fache von 7,4 % auf 20,8 %. In 20,1 % der Meldungen wurde Technik, in 27,7 % Medizinprodukte erwähnt. Medikamente waren in 10,7 % der Meldungen Gegenstand der Zwischenfälle. Aus dem perioperativen Umfeld wurden 47,8 % der stationären Zwischenfälle berichtet, 24,6 % aus den Bereichen Intensivstation/Aufwachraum. Das CIRS-Team des BDA analysierte und kommentierte 36,1 % der Fälle .

**Schlussfolgerung:**

Die Analyse liefert Erkenntnisse für die Gestaltung zukünftiger Melderichtlinien und Anwenderschulungen. Insbesondere der Häufung „sonstiger“ Meldungen, welche nicht den Kriterien einer CIRS-Meldung entsprechen, sollte künftig Rechnung getragen werden.

## Abstract (en)

**Background:**

Critical incident reporting systems (CIRS) serve to sensitize organizations and individuals to unknown events relevant to patient safety and therefore help in developing safer systems; however, the use and impact of these systems in healthcare has recently been questioned for a variety of reasons, among them unclear and imprecise reporting criteria. Some authors claim that fundamental aspects of successful CIRS have been misunderstood, misapplied or entirely missed during the adaptation to the healthcare context. The aim of this study was the analysis of all reports accumulated over 10 years in the German system CIRSmedical Anesthesiology (CIRS-AINS) as a basis for improved reporting guidelines, user training and generation of further hypotheses.

**Methods:**

In a retrospective analysis all reports from CIRS-AINS entered between April 2010 and June 2019 were analyzed for structure and content.

**Results:**

A total of 6013 reports were filed consisting of 3492 incidents (58.1%), 1734 near misses (28.8%) and 787 others (13.1%). Those other reports contained 21 interpersonal conflicts (0.4%), 102 general complaints (1.7%), 89 stress or workload complaints (1.5%) and 575 reports that did not contain any critical incident or safety-related content (9.6%). Since 2015 these other reports have increased 2.8-fold from 7.4% to 20.8%. Of the reports 20.1% contained information about technical problems and 27.7% about certified medical devices. Medication was mentioned in 10.7% of reports, 47.8% of inpatient incidents concerned the perioperative setting, 24.6% were reported from intensive care units (ICU) and postanesthesia care units (PACU). Of the cases 198 (3.3%) explicitly mentioned communication issues, 346 cases (5.8%) concerned incomplete or inadequate documentation involving orders, blood products or laboratory tests. Of the reports 36.1% were analyzed and commented on by the CIRS team of the German Society of Anesthesiologists (BDA).

**Conclusion:**

The analysis provides insights into reporting practices and can influence both reporting guidelines as well as user training. Report format, content and context are of utmost importance for further analysis: A distinction has to be made between reports that contain locally rational information and cannot be understood without further context and reports that may help inform about patient safety activities on a national level. Especially in light of the limited resources for incident analysis, the content should be critically reflected upon by the user when submitting a report to support a wise allocation of available capacities. In this respect, the increase of non-CIRS reports has to be considered in the future implementation of nationwide IRS. Also, it has to be questioned whether adequate alternative means of communication for these non-CIRS reports exist. The majority of reports were made by physicians, which is in contrast to international experiences with increased engagement of nursing staff and underlines the need for increased interprofessional collaboration with incident reporting and analysis activities in Germany. Reports containing workload complaints, while constituting important signals on a local level, usually fail to address the idea of learning from others inherent to the philosophy of national IRS.

Seit den wegweisenden Publikationen des Institute of Medicine (IOM) „To err is human“ aus dem Jahre 1999 [7] und des National Health Service (NHS) „An organisation with a memory“ [3] aus dem Jahre 2000, in welchen auf die zentrale Rolle eines „Incident-reporting“-Systems (IRS) in Bezug auf die Patientensicherheit verwiesen wurde, sind IRS weltweit in der stationären und in der ambulanten Gesundheitsversorgung zu den am meisten verbreiteten Strategien zur Verbesserung der Patientensicherheit geworden. Wenige Jahre nach den genannten Publikationen unterstrich auch die Weltgesundheitsorganisation (WHO) den zentralen Stellenwert von Berichtssystemen für das Lernen aus Zwischenfällen und Fehlern [17].

## Hintergrund

In Deutschland wurde 2013 das Gesetz zur Verbesserung der Rechte von Patientinnen und Patienten verabschiedet. Der Gemeinsame Bundesausschuss hat in Folge die Einrichtung interner und einrichtungsübergreifender Fehlermeldesysteme verpflichtend gemacht und die Anforderungen an solche Systeme präzisiert [5]. Das Betreiben einrichtungsübergreifender Fehlermeldesysteme sollte mit einem finanziellen Anreiz versehen werden, sodass davon ausgegangen werden kann, dass diese Systeme in Krankenhäusern und im ambulanten Sektor großflächig verfügbar sind.

Während sich vielerorts die Verantwortlichen dieser Systeme noch um deren soziokulturelle Aspekte bemühen (z. B. Akzeptanz, Meldeverhalten) und auf eine breitere Beteiligung der Mitarbeiter hoffen, mehren sich in der wissenschaftlichen Literatur zu Incident reporting zunehmend Stimmen, die auf grundlegende Defizite in der Umsetzung von Incident-reporting-Systemen (IRS) im Gesundheitswesen hinweisen. Sie postulieren, dass diese Systeme in der gegenwärtigen Konzeptionierung nicht die Hoffnungen erfüllen können, die in sie gesetzt werden [8, 9]. Die grundlegende Kritik lautet, dass im Gesundheitswesen ein erfolgreiches Modell aus anderen soziotechnischen Systemen (v. a. der zivilen Luftfahrt) kopiert werden soll, ohne gleichzeitig die für den Erfolg notwendigen finanziellen und personellen Ressourcen vorzuhalten. Das erschwert den Aufbau einer grundlegenden sozialen Infrastruktur aus kritischem Dialog mit dem Melder, umfassender Untersuchung und rascher Ableitung von konstruktiven Maßnahmen. Laut der Kritik resultiert dies in einem System, in dem fundamentale Aspekte des Incident reporting missverstanden und falsch angewendet werden oder gänzlich fehlen [1, 9, 10]. Incident-reporting-Systeme im Gesundheitswesen, so die Kritiker, sind dadurch charakterisiert, dass die Quantität der Berichte anstatt der Qualität der resultierenden Investigation als Maß für die Effizienz des Systems herangezogen wird [2, 10]. Der Mangel an sinnvollen, evidenzbasierten Reaktionen ist auch darauf zurückzuführen, dass die Berichte oft zu wenig Informationen beinhalten, um den gemeldeten Vorfall analysieren zu können [15].

Für die deutschsprachige Anästhesiologie stellt sich die Frage, ob diese in der Literatur vielfach geäußerte Fundamentalkritik einer ungenügenden Qualität von Meldungen auch auf CIRS-AINS, das IRS des Berufsverbands Deutscher Anästhesisten (BDA) und der Deutschen Gesellschaft für Anästhesiologie und Intensivmedizin (DGAI) zutrifft. Darüber hinaus ist in den letzten Jahren bei den Mitarbeitern des Anonymisierungs- und Analyseteams von CIRS-AINS der Eindruck entstanden, dass zunehmend Meldungen abgegeben werden, die keinen konkreten Bezug zu einem sicherheitsrelevanten Vorfall haben und deren Inhalt eher den Charakter einer Beschwerde oder Überlastungsanzeige trägt. Derartige Meldungen können jedoch nur bedingt zur Verbesserung der Patientensicherheit beitragen. Ziel der vorliegenden Arbeit war es daher, alle Meldungen in CIRS-AINS seit dessen Beginn im Jahr 2010 dahingehend zu prüfen, ob dieser subjektive Eindruck durch eine Analyse der Daten bestätigt werden kann.

## Studiendesign

In einer retrospektiven Datenbankanalyse wurden alle Meldungen in CIRS-AINS aus dem Zeitraum von April 2010 bis Juni 2019 analysiert. Der Inhalt jeder Meldung wurde dahingehend überprüft, ob in ihm ein Zwischenfall oder Beinahe-Zwischenfall berichtet wurde, oder ob interpersonelle Konflikte, Beschwerden oder Überlastungsanzeigen Gegenstand der Meldung waren. Meldungen, die keiner dieser Kategorien zugeordnet werden konnten, wurden gesondert erfasst (für eine Übersicht: Tab. 1). Alle Elemente des CIRS-AINS-Meldebogens (z. B. Ort, Zeitpunkt, Fachgebiet, Berufserfahrung, Berufsgruppe, ASA-Klassifikation etc.; Tab. 2) sowie die Vollständigkeit der Meldung wurden deskriptiv erfasst und der Umfang der Freitextmeldungen analysiert.

**Table 1:** 

Kategorie	Beschreibung	Ursprüngliches Element von CIRS
1	Zwischenfall („incident“)	Ja
2	Beinahe-Zwischenfall („near-miss“)	Ja
3	Konflikt	Nein
4	Beschwerde	Nein
5	Überlastungsanzeige	Nein
6	Sonstige	Nein

**Table 2:** 

CIRS-AINS-Meldebogen	
Zuständiges Fachgebiet	
Wo ist das Ereignis passiert	Krankenhaus/Rettungsdienst/Praxis
	Einleitung/Ausleitung/Transport/Schmerzambulanz/Notfallaufnahme/OP/ITS/IMC/AWR/Funktions- bzw. Diagnostikraum/Notfall-Team-Einsatz/Normalstation/PM-Ambulanz/Akutschmerzdienst/anderer Ort
Tag des berichteten Ereignisses	Wochentag
	Wochenende/Feiertag
Welche Versorgungsart	Routinebetrieb/Notfall
ASA-Klassifizierung (Vor dem Ereignis)	ASA I–V
Patientenzustand (Nur sofern relevant oder interessant)	(Optionaler Freitext)
Wichtige Begleitumstände (Nur sofern relevant oder interessant)	(Optionaler Freitext)
War ein Medizinprodukt beteiligt?	Ja/Nein
Fallbeschreibung (Was, warum, Kofaktoren, Maßnahmen, Effektivität, Verlauf, Epikrise)	(Optionaler Freitext)
Was war besonders gut (Hat zur Abschwächung des Ereignisses oder zur Verhinderung eines Patientenschadens geführt)	(Optionaler Freitext)
Was war besonders ungünstig (Hat zur Verschlimmerung des Ereignisses oder zur Verstärkung des Patientenschadens beigetragen)	(Optionaler Freitext)
Eigener Ratschlag („take-home message“) (Welche Maßnahmen könnten ein derartiges Ereignis in Zukunft verhindern, unwahrscheinlicher machen oder dessen Folgen abmindern?)	(Optionaler Freitext)
Wie häufig tritt ein Ereignis dieser Art in Ihrer Abteilung auf?	Fast täglich/jede Woche/jeden Monat/mehrmals pro Jahr/selten/nur dieses Mal
Wer berichtet (Optionale Angabe)	Ärztin/Arzt
	Pflegekraft
	Andere (z. B. MTA, PTA, Rettungsassistent, Rettungssanitäter, Notfallsanitäter, Techniker o. Ä.)
Ihre Berufserfahrung	Bis 5 Jahre
	Über 5 Jahre

^a^https://www.cirsmedical.ch/AINS/m_files/cirs.php?seitennr=AINS

Die deskriptive Datenanalyse erfolgte unter Angabe von absoluten und relativen Häufigkeiten bzw. deren Mittelwerten/Medianen und Standardabweichungen (Microsoft Excel for Mac Version 15.32, Microsoft Corp., Redmont, WA, USA).

Die Studie wurde durch die Ethikkommission der Medizinischen Fakultät der Universität Heidelberg genehmigt (S-775/2018).

## Ergebnisse

**Figure 1:**
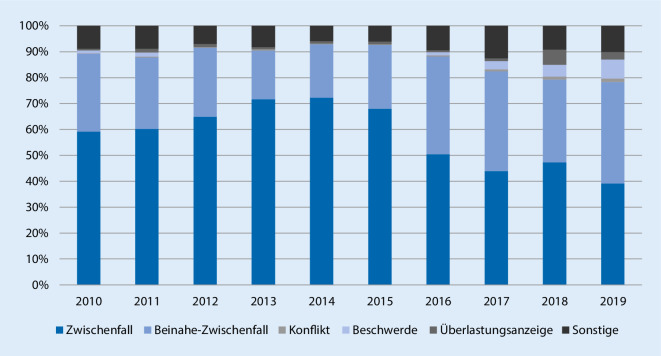

Aktuell nehmen 94 Kliniken an CIRS-AINS teil. Die Nutzungsstatistiken zeigen eine relativ konstante Zahl von ca. 500 bis 600 Meldungen/Jahr seit 2011. Im betrachteten Zeitraum wurden 6013 CIRS-Fälle gemeldet. Aufgrund von Umstrukturierungen der CIRS-AINS-Datenbank in der Anfangszeit des Systems konnte auf die Fälle der Monate April 2010, Juli 2010 und Dezember 2011 nicht zugegriffen werden, sodass diese nicht in die Analyse einbezogen wurden. 819 Fälle konnten keinem Zeitraum (Monat/Jahr) zugeordnet werden. Monatlich wurden in CIRS-AINS durchschnittlich 31 Fälle gemeldet (SD 11). Die Fälle setzen sich aus 3492 Zwischenfällen (Kategorie 1; 58,1 %), 1734 Beinahe-Zwischenfällen (Kategorie 2; 28,8 %) und 787 weiteren Meldungen (Kategorien 3–6; 13,1 %) zusammen. Die Gruppe der weiteren Meldungen setzte sich aus 21 Konfliktmeldungen (Kategorie 3; 0,4 %), 102 Beschwerden (Kategorie 4; 1,7 %), 89 Überlastungsanzeigen (Kategorie 5; 1,5 %) sowie 575 sonstigen Meldungen, die keine CIRS-Fälle darstellen (Kategorie 6; 9,6 %), zusammen. Eine Übersicht der Meldungsverteilung nach Kategorien und Jahr bietet Abb. 1. Seit 2015 zeigt sich eine stetige Zunahme an Meldungen der Kategorien 3–6 um das 2,8-Fache von 7,4 % auf 20,8 %.

Bei 4179 Meldungen (69,5 %) waren die Versorgungsbedingungen bekannt (*n* = 4013 [66,7 %] stationär, *n* = 50 [0,8 %] ambulant, *n* = 116 [1,9 %] präklinisch). 45,2 % (*n* = 1813) der stationären Zwischenfälle wurden aus dem perioperativen Umfeld berichtet, 26,2 % (*n* = 1052) aus den Bereichen Intensivstation/IMC und Aufwachraum. 282 Fälle (7,0 %) ereigneten sich auf Normalstationen, 135 (3,4 %) in Funktions‑/Diagnostikbereichen (z. B. CT, MRT, Herzkatheter etc.). Bei 335 Fällen (8,4 %) fehlten Angaben zu Umgebungsbedingungen bzw. Ort.

Die überwiegende Anzahl der Fälle stammt aus der Anästhesie (*n* = 5280, 87,8 %). Da seit Dezember 2011 CIRS-AINS von Kliniken auch einrichtungs- und fachübergreifend verwendet werden konnte, finden sich in CIRS-AINS auch Meldungen, die aus anderen Fachgebieten stammen. Eine Übersicht über die Meldungen, aufgeteilt nach beteiligten Fachrichtungen, bietet (Tab. 3).

**Table 3:** 

Fachgebiet	Anzahl der Meldungen	Anteil (%)
Anästhesie	5280	87,8
Allgemeinmedizin	16	0,3
Augenheilkunde	3	0,0
Chirurgie	175	2,9
Hals-Nasen-Ohren-Heilkunde	9	0,1
Innere Medizin	177	2,9
Neurologie	13	0,2
Pädiatrie	9	0,1
Unfallchirurgie	18	0,3
Urologie	9	0,1
Keine Angabe	304	5,1

**Figure 2:**
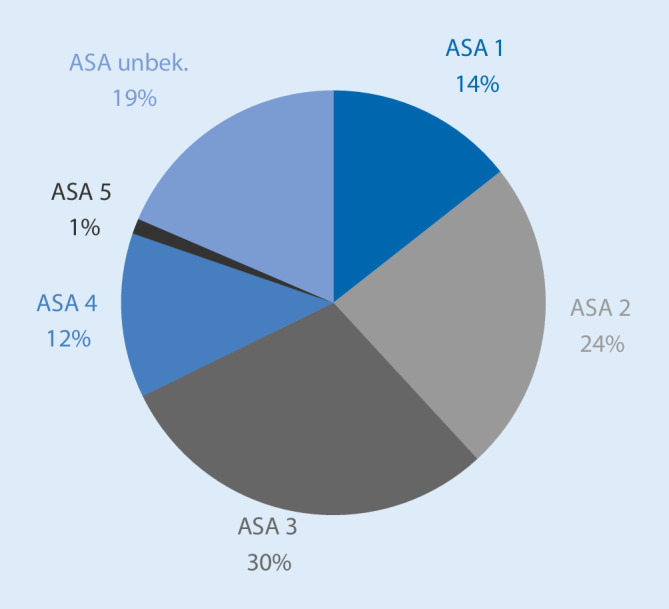

**Figure 3:**
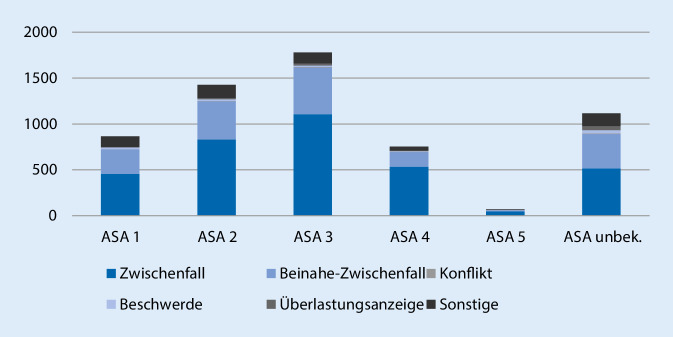

4538 Fälle (75,5 %) wurden aus dem Routinebetrieb gemeldet, 1194 (19,6 %) aus der Notfallversorgung, bei 281 Fällen (4,7 %) wurden keine Angaben gemacht. Die überwiegende Mehrzahl der Fälle ereignete sich wochentags (*n* = 4538 [75,5 %]), 1194 Fälle (19,9 %) ereigneten sich am Wochenende, bei 281 Fällen (4,7 %) erfolgte keine Angabe. Die Verteilung nach ASA-Klassifikation zeigt Abb. 2, die Verteilung der Kategorien innerhalb der ASA-Klassifikation zeigt Abb. 3.

In 1207 (20,1 %) Meldungen wurden Technik, in 1665 (27,7 %) Medizinprodukte erwähnt. Medikamente waren in 646 (10,7 %) der Meldungen Gegenstand der Zwischenfälle. In 198 Fällen (3,3 %) wurden explizit Probleme in der Kommunikation gemeldet. Unvollständige, mangelhafte, vertauschte oder unleserliche Dokumentation wurde in 346 Fällen (5,8 %) erwähnt; dies betraf sowohl Anordnungen als auch Laboretiketten oder Chargendokumentation von Blutprodukten. 63 Fälle (1,0 %) beschreiben Zwischenfälle mit Periduralkathetern oder zentralen Venenkathetern; hierbei wurden in 55 Fällen Verwechslungen und damit einhergehende Fehlkonnexionen beschrieben (i.v.-Applikation von Lokalanästhetika bzw. intrathekale Applikation von i.v.-Medikation). In 8 Fällen wurden Substanzen i.v. verabreicht, die für die Applikation via Magensonde bestimmt gewesen waren.

**Figure 4:**
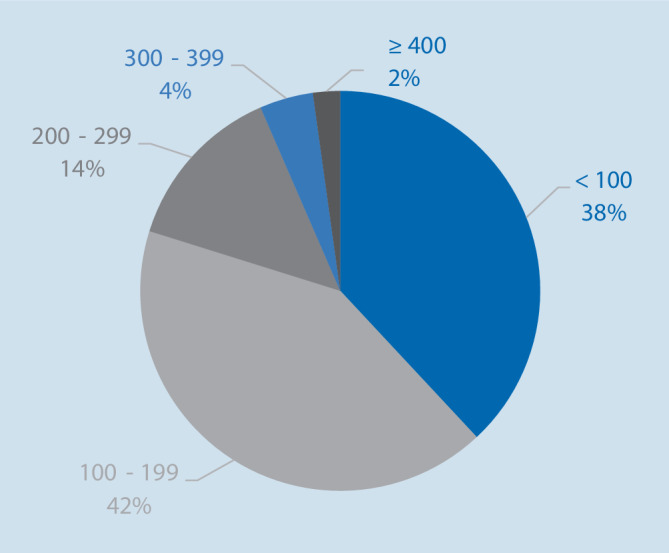

Die Mehrzahl der Fälle wurde von Mitarbeitern mit >5 Jahren Berufserfahrung berichtet (*n* = 4201 [69,9 %]), 1143 (19,0 %) von jüngeren Mitarbeitern. Ärztliche Mitarbeiter meldeten 3990 Fälle (66,4 %), Mitarbeiter der Pflege 1593 Fälle (26,5 %). Im Median umfassten die Fallbeschreibungen 121 Wörter (±95, für die Verteilung: Abb. 4). 3669 Meldungen enthielten Details zum Patientenzustand (61,0 %), 2797 zu wichtigen Begleitumständen (46,5 %).

**Figure 5:**
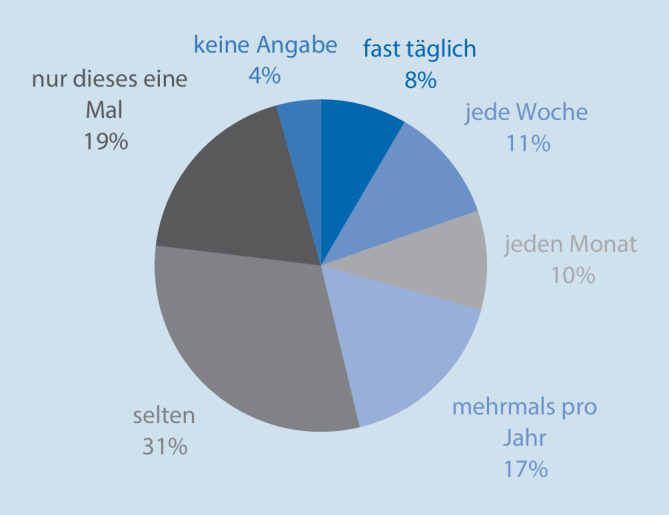

Positive Aspekte wurden durch die Meldenden in 3929 Fällen hervorgehoben (65,3 %), negative in 4184 (69,6 %). Eigene Ratschläge oder Anregungen für Verbesserungen wurden in 4976 Fällen (82,2 %) übermittelt. Eine Übersicht über die durch die Meldenden geschätzte Auftretenswahrscheinlichkeit bietet Abb. 5.

**Figure 6:**
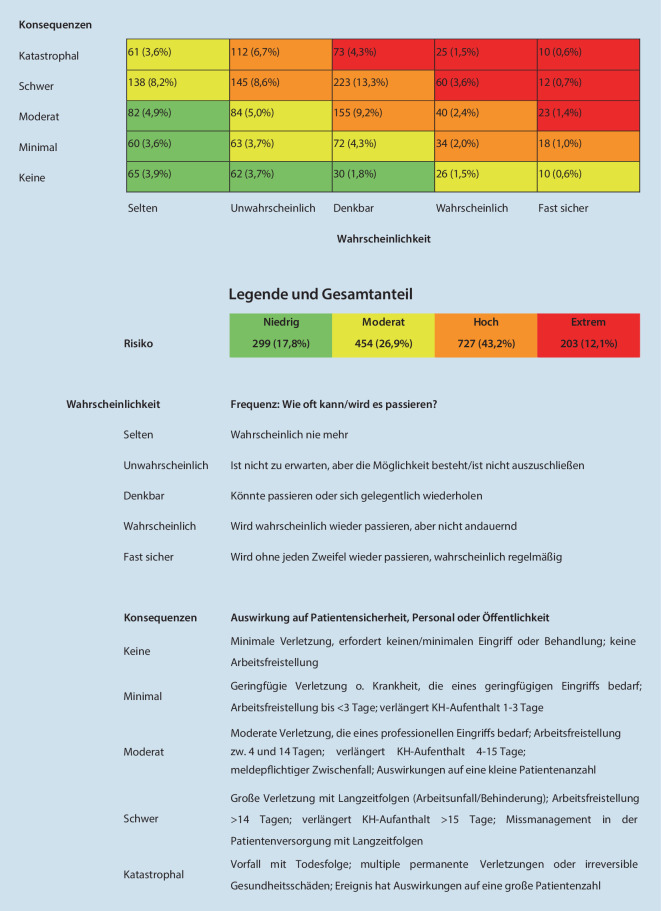

36,1 % der Fälle (*n* = 2172) wurden vom CIRS-AINS-Analyseteam bearbeitet und kommentiert. Bei ausreichender Datengrundlage der Meldung erfolgte zur Risikoabschätzung des Ereignisses eine Einstufung in die Risikomatrix (*n* = 1683; Abb. 6).

## Diskussion

In der vorliegenden Arbeit konnten aus 6013 Fällen Erkenntnisse über Meldeverhalten, Struktur und Inhalt der Fallbeschreibungen eines abteilungsübergreifenden CIRS gewonnen werden.

Der überwiegende Teil an Meldungen in CIRS-AINS wurde von Ärztinnen und Ärzten eingegeben, mit einer deutlich geringeren Beteiligung von Pflegekräften. Diese Relation im Meldeverhalten ist bemerkenswert, da international Berichtsysteme überwiegend von Pflegekräften genutzt werden und Ärzte sich kaum daran beteiligen [6, 10, 11]. Bei einigen Systemen scheint ein Grund für dieses ungleiche Meldeverhalten darin zu liegen, dass die Ärzteschaft nicht ausreichend in die Konzeptionierung und Implementierung der jeweiligen Systeme eingebunden gewesen war und das Berichten von schwerwiegenden Ereignissen daher nicht im Bereich ihrer Verantwortung sah [10]. Somit könnte die rege Beteiligung von Ärzten in CIRS-AINS u. a. darin begründet liegen, dass sowohl die Initiative als auch die Implementierungsstrategie von einem ärztlichen Berufsverband (BDA) in Kooperation mit dem Ärztlichen Zentrum für Qualität in der Medizin (ÄZQ) ausging und in der Anfangsphase primär Ärzte als Ansprechpartner hatte.

Die Hälfte der gemeldeten Ereignisse wurden vom Melder als seltenes oder erstmaliges Ereignis („nur dieses eine Mal“) eingestuft, wodurch sie in der erlebten Realität der Meldenden den Charakter eines neuen, bisher nicht bekannten und potenziell gefährlichen Risikos darstellen, dem in der jeweiligen Arbeitsumgebung Rechnung getragen werden sollte. Bedenklich erscheint die Tatsache, dass unabhängig von „sonstigen“ Inhalten fast ein Drittel der Berichte echte Sicherheitsrisiken thematisieren, die sich nach Einschätzung der Melder monatlich (10 %), wöchentlich (11 %) oder fast täglich (8 %) wiederholen. Die Meldung dieser Ereignisse demonstriert zwar ein Risikobewusstsein aufseiten des Melders, dokumentiert aber gleichzeitig eine gewisse Vulnerabilität der anästhesiologischen Patientenversorgung aufgrund ungenügender organisationaler oder struktureller Änderungen in Reaktion auf bekannte Risiken: Die Gefahrenquellen sind bekannt und können sich dennoch mit großer Regelmäßigkeit im klinischen Alltag realisieren. Eine detailliertere Analyse zu Hintergründen und der lokalen (Ir‑)Rationalität im Umgang mit den jeweiligen Ereignissen bleibt den lokalen CIRS-Teams vorbehalten und unterliegt all den damit verbundenen Möglichkeiten und Limitationen.

Viele CIRS-Meldungen zeichneten sich durch eine sehr kurze Fallbeschreibung aus; in 2280 Berichten (38 % aller Meldungen) beschränkten sich die Melder auf weniger als 100 Worte, um den für sie sicherheitsrelevanten Sachverhalt zu schildern. In der Sicherheitsforschung wird die Wichtigkeit einer detaillierten Schilderung unterschiedlich gedeutet. So gibt es Stimmen, die eine Meldung mit sicherheitsrelevantem Inhalt lediglich als Anfangspunkt eines investigativen Prozesses sehen, welcher durch ausgebildetes Personal erfolgen sollte. Da sich dieses Analyseteam vor Ort ein detailliertes Bild des Vorfalls verschaffen und die Fakten mit geschultem Blick interpretieren wird, sind weder eine umfangreiche Meldung noch eine Analyse durch den Berichtenden notwendig [14]. Diese Sichtweise spiegelt die in anderen soziotechnischen Systemen vorhandene soziale Infrastruktur aus kritischem Dialog mit dem Melder, umfassender Untersuchung und rascher Ableitung konstruktiver Maßnahmen wider. Für eine Realisierung im Gesundheitswesen sind jedoch in der Regel weder die notwendigen finanziellen und personellen Ressourcen noch eine zugrunde liegende offene, wertschätzende Fehlerkultur vorhanden. Andere Autoren wiederum kommen zu dem Ergebnis, dass ein wesentliches Manko der meisten Reporting-Systeme sowohl im Umfang als auch in der Qualität der eingegebenen Informationen liegt. Da die Meldungen häufig zu wenig detaillierte Angaben sowohl zum Ereignis als auch zu ursächlichen oder beitragenden Faktoren enthalten, ist eine systematische Analyse oft nicht möglich [15].

Die oftmals fehlenden finanziellen und personellen Ressourcen haben die Diskussion neu belebt, ob es im Kontext aktueller CIRS im Gesundheitswesen Sinn macht, Beinahe-Zwischenfälle zu melden. Beinahe-Zwischenfälle beinhalten zwar relevante Informationen über offensichtliche Gefährdungsquellen, treten häufiger auf als Schadensfälle, sind unproblematischer zu melden und werden in ihrer Analyse nicht durch den Rückschaufehler verzerrt [1]; da aber die meisten Organisationen aufgrund fehlender Ressourcen oder mangelnder Bereitschaft Meldungen von Zwischenfällen nicht zeitnah und adäquat analysieren, so die Kritiker, erscheint es von fraglichem Wert, wenn Melder eine noch größere Anzahl an Beinahe-Zwischenfällen mitteilen, auf die dann gar nicht reagiert wird [4]. Diese Argumentation erscheint für CIRS-AINS jedoch nur bedingt relevant: Bei aktuell 94 teilnehmenden Kliniken und 500 bis 600 Meldungen/Jahr fallen pro Klinik jährlich durchschnittlich 6 bis 7 Meldungen an, sodass diese Anzahl auch mit geringeren Ressourcen gut zu bewältigen sein dürfte. Eine differenziertere Betrachtung erfordert vielmehr der qualitative Aspekt der CIRS-Berichte: Bei annähernd konstanten Meldezahlen pro Jahr zeigt sich seit 2015 eine deutliche Zunahme an Meldungen, bei deren Inhalt es sich um Schilderungen interpersoneller Konflikte, um Beschwerden hinsichtlich als sicherheitsrelevant eingestufter Entscheidungen anderer und um Beschreibungen von Arbeitssituationen mit chronischer personeller Unterbesetzung handelt, ohne dass dabei auf ein Ereignis mit potenzieller oder tatsächlicher Patientenschädigung Bezug genommen wird. Eine mögliche Interpretation dieser Entwicklung besteht darin, dass CIRS-AINS als geeignetste oder, in Ermangelung anderer Wege, als einzige Möglichkeit zur innerklinischen (kritischen) Kommunikation gesehen wird. Möglicherweise verbindet der Melder mit der Eingabe die Hoffnung, dass eine Schilderung von Missständen (z. B. chronische personelle Unterbesetzung) in CIRS-AINS Ressourcen mobilisieren kann, welche die eigene Organisation augenblicklich nicht zur Verfügung stellt. Von entscheidender Bedeutung ist, sowohl in Bezug auf Anzahl als auch Art der Meldungen, die lokale CIRS-Gruppe. Wenn diese gut vernetzt ist, über Ressourcen verfügt und Unterstützung durch die Leitungsebene erfährt, kann auf Meldungen reagiert werden, die zwar im abteilungsübergreifenden IRS keinen Erkenntnisgewinn bieten (z. B. Überlastungsanzeigen), durchaus aber auf lokaler Ebene ein wichtiges Signal darstellen und sicherheitsrelevante Änderungen anstoßen können. Derartig emergente Funktionen weichen zwar von der ursprünglichen Idee eines IRS ab, schließen aber möglicherweise Lücken im sonstigen Angebot zum Dialog zwischen Mitarbeitern und Führungskräften an teilnehmenden Kliniken.

Ganz sicher jedoch ist diese Entwicklung mit der grundlegenden Philosophie assoziiert, welche Incident-Bericht-Systeme im Gesundheitswesen auszeichnet. Die resultierenden Prinzipien und Praktiken unterscheiden sich in vielen Aspekten teilweise erheblich von denen des Incident reporting in anderen sicherheitskritischen Industrien (Tab. 4).

**Table 4:** 

Zentrale Prinzipien in anderen Industrien	Übliche Herangehensweise im Gesundheitswesen
Der Fokus liegt auf dem Berichten von Vorfällen, welche ernst zu nehmende, spezifische oder überraschende Einsichten in die Systemsicherheit bieten	Es wird ermutigt, jeden nur denkbaren Vorfall zu berichten, der in irgendeiner Weise Sicherheitsrelevanz haben könnte
Nach Möglichkeit sollte das Berichtssystem nicht mit Meldungen überflutet werden, damit sichergestellt wird, dass jeder einzelne Bericht gründlich bearbeitet werden kann	Eine hohe Anzahl an Meldungen wird als Erfolg gefeiert, und das Ziel sollte darin bestehen, dass die Zahl der Berichte ständig zunimmt
Incident reports sollen dafür verwendet werden, dass signifikante, neue oder sich entwickelnde Risiken identifiziert und priorisiert werden können	Incident reports werden quantifiziert, gezählt und kategorisiert, um Entwicklungstrends zu erfassen
Taxonomien werden pragmatisch eingesetzt, um die grundlegende Analyse, das Ergreifen von Maßnahmen und eine retrospektive Maßnahmenkontrolle zu unterstützen	Von Taxonomien kann man erwarten, dass sie komplexe Realitäten zutreffend wiedergeben und erklären
Es wird sichergestellt, dass Berichtssysteme durch eine von der Organisation unabhängige Gruppe betreut und koordiniert werden	Meldungen werden an direkte Vorgesetzte oder andere Führungspersonen innerhalb der Organisation weitergeleitet
Das Berichten von Vorfällen stellt eine Komponente innerhalb einer Vielzahl an Konversationen und Aktivitäten dar, die auf Sicherheiten und Risiken fokussieren	Incident reporting stellt für viele Organisationen die sichtbarste Aktivität im Bereich von Sicherheit dar

Insbesondere die Sichtweise, dass die Anzahl an Meldungen als Indikator für Sicherheit in den jeweiligen Organisationen gewertet werden kann, teilen viele dieser Industrien nicht. Bei ihnen beschreiben die Berichte vorrangig diejenigen Ereignisse, welche auf sehr spezifische, ernst zu nehmende, und bis dato nicht bekannte Risiken hinweisen [9, 16]. Mithilfe dieser Fokussierung soll angesichts begrenzter Ressourcen erreicht werden, dass die Aufarbeitung der einzelnen Meldung adäquat erfolgen kann, um Risiken zeitnah zu identifizieren und priorisieren. Diese explizite Fokussierung ist in CIRS des Gesundheitswesens in der Regel nicht zu finden. Vielmehr werden in den verschiedenen Systemen sehr allgemeine und vieldeutige Kriterien aufgeführt, die dem Melder viel Spielraum für persönliche Interpretation lassen (Infobox 1). Hieraus resultiert eine Fülle an Meldungen mit wenig relevanten Inhalten anstelle einer kleinen Menge an Berichten, die kritische Aspekte der Patientensicherheit thematisieren [10].

Auch CIRS-AINS ist von dieser grundlegenden Kritik an Reporting-Systemen im Gesundheitswesen betroffen. Die Arbeitsgruppe Incident Reporting des Forums Qualitätssicherung und Ökonomie des BDA und der DGAI vertrat bei der Konzeptionierung von CIRS-AINS die Position, dass „in einem modernen IRS alle sicherheitsrelevanten Ereignisse gesammelt werden, die zu einer Gefährdung des Patienten führen können oder dazu geführt haben“ [13]. Die „goldene Regel“ des Reporting, so ist auf der Webseite von www.cirs-ains.de zu lesen, lautet: „Berichten Sie alles, was Sie gerne vorher gewusst hätten“. Rückblickend kann vermutet werden, dass der Fokus hierbei weniger auf der Qualität der dadurch generierten Meldungen lag als vielmehr auf einer möglichst raschen und umfassenden Beteiligung von Pflegekräften und Ärzten am neu etablierten System.

Für die Sicherheitsforschung gilt als Erfolgskriterium eines IRS nicht die Anzahl der eingegebenen Meldungen, sondern das Ausmaß der aus den Meldungen resultierenden systemischen Veränderung und Risikoreduktion [12]. Die Tatsache, dass diese oftmals schwierig zu beurteilen oder gar zu messen sind, mag erklären, warum Meldezahlen willkommene Surrogatparameter darstellen.

### Infobox 1 Beispiele für Meldekriterien unterschiedlicher CIRS

- „Alle sicherheitsrelevanten Ereignisse, die in der Medizin auftreten, können von Mitarbeiterinnen und Mitarbeitern des Gesundheitswesens berichtet werden“ (www.cirsmedical.de)
- „Als ‚zu berichtende Ereignisse‘ gelten alle Fehler, Risiken, kritischen Ereignisse und Beinahe-Schäden in der Versorgung der Patienten, Bewohner bzw. Klienten, wenn zum Zeitpunkt des Berichtens kein Schaden des Patienten, Bewohners bzw. Klienten durch das Ereignis bzw. das Risiko erkennbar ist. Es ist zudem sinnvoll, die Mitarbeiter explizit zu motivieren, auch über die erfolgreiche Bewältigung von Fehlern und Lösungsansätze für kritische Situationen zu berichten“ [13]
- „Im Prinzip sollen alle ‚sicherheitsrelevanten Ereignisse‘ berichtet werden. Oft wird auch von ‚unerwünschten Ereignissen‘ gesprochen“ [15]
- „Berichten Sie alles, was Sie gerne vorher gewusst hätten“ (www.cirs-ains.de)
- „Im Rahmen eines CIRS werden alle Informationen gemeldet, die zur Erhöhung der Patientensicherheit beitragen. Dabei muss es sich nicht ausschließlich um tatsächliche Fehler oder unterwünschte Ereignisse handeln“ (www.cirs-health-care.de)
- „Sie dürfen Fälle aus dem kompletten Bereich Ihres Alltags eingeben, die mit Ihrem medizinischen Studienfach zu tun haben. Dies können Situationen in der Praxis oder Klinik sein, Team-Kommunikationssituationen, Lehrveranstaltungen, aber auch Fehler in Lehrmaterialien oder bei Lehr- und Prüfungsmethoden u. v. m“ (https://www.cirsmedical.de/impp)

**Figure 7:**
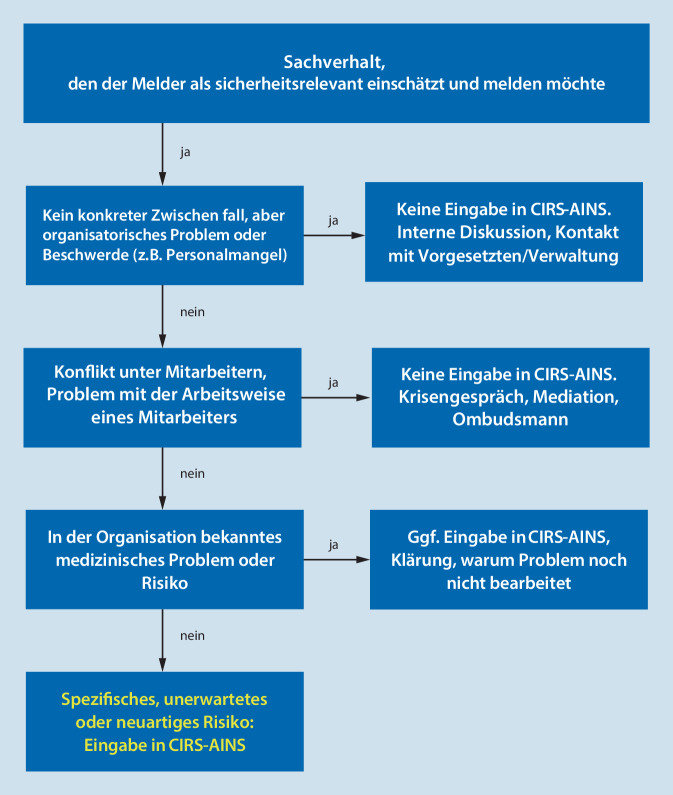

Es erscheint daher sinnvoll, neue Impulse und Argumente in die Diskussion um die Nutzung von IRS im Allgemeinen und die Eignung einer Meldung im Speziellen einzubringen. Nur so kann das System langfristig und nachhaltig einen relevanten Beitrag zum besseren Verständnis aktueller Herausforderungen für die Patientensicherheit leisten. Auf institutioneller Ebene erfordert jedes IRS Unterstützung durch die Klinikleitung, welche neben personellen Ressourcen vorrangig in der Bereitschaft zu Reaktion und Veränderung besteht. Ohne derartig essenzielle Voraussetzungen erfüllt ein IRS lediglich eine gesetzlich vorgeschriebene Alibifunktion. Dem individuellen Melder bietet Abb. 7 Unterstützung bei der Frage, wie mit einer durch ihn wahrgenommenen, potenziellen Patientengefährdung verfahren werden kann.

### Limitationen

Eine der vorrangigen Limitationen der vorliegenden Arbeit liegt in der Unmöglichkeit der Kontaktaufnahme mit den Verfassern der Meldungen aufgrund der Anonymisierung der Datenbankeinträge. Hierdurch sind konkretere Analysen zu Meldeverhalten und -motivation im Zusammenhang mit den vorliegenden Daten unmöglich. Darüber hinaus können aus den vorliegenden Daten keine Aussagen dazu getroffen werden, wie die Informationen aus dem System vor Ort verwendet wurden, um die Patientensicherheit im Behandlungsprozess zu verbessern oder organisationales Lernen und Veränderungen anzustoßen. Es erscheint jedoch eine legitime Schlussfolgerung, dass es in Abhängigkeit von Inhalt und Umfang der Meldung schwierig werden kann, sinnvolle und umfassende Veränderungen daraus abzuleiten.

## Fazit für die Praxis

- In der Interpretation der Meldungen muss zwischen örtlich und abteilungsübergreifend relevantem Inhalt unterschieden werden. So kann eine Meldung für das lokale CIRS-Team von großer Signifikanz sein, für Leser aus anderen Institutionen aber schwer bis nicht verständlich sein.
- Im Sinne der interprofessionellen Zusammenarbeit scheint es dringend geboten, Pflegende stärker in alle Aspekte der CIRS-Arbeit einzubinden. So können diverse Sichtweisen des klinischen Alltags repräsentativer abgebildet werden.
- Überlastungsanzeigen, verstanden als die Schilderung einer personellen Unterbesetzung ohne gleichzeitigen Bezug zu einem Ereignis mit Patientengefährdung, bilden in einem nationalen System wie CIRS-AINS sicherlich allgemeine Probleme des Gesundheitssystems ab, erreichen aber keine Verbesserung der Patientensicherheit gemäß dem Gedanken „Lernen von anderen“.
- Für das organisationale Lernen ist die inhaltliche Qualität der Meldungen eine wesentliche Voraussetzung, weswegen jeder Nutzer beim Verfassen einer Meldung diese kritisch auf Eignung für ein IRS, Verständlichkeit und Qualität überprüfen sollte. Nur auf dieser Grundlage können knappe Ressourcen zur Verbesserung der Versorgungsqualität adäquat und zielgerichtet eingesetzt werden.

## Notes

Für diesen Beitrag wurden von den Autoren keine Studien an Menschen oder Tieren durchgeführt. Für die aufgeführten Studien gelten die jeweils dort angegebenen ethischen Richtlinien.

## References

[1] Barach P, Small SD (2000). Reporting and preventing medical mishaps: lessons from non-medical near miss reporting systems. BMJ.

[2] Battles JB, Stevens DP (2009). Adverse event reporting systems and safer healthcare. BMJ Qual Saf.

[3] Department of Health (2000). An organisation with a memory: report of an expert group on learning from adverse events in the NHS chaired by the Chief Medical Officer.

[4] Edmondson AC (2004). Learning from failure in health care: frequent opportunities, pervasive barriers. BMJ Qual Saf.

[5] Gemeinsamer Bundesausschuss (2016). Bestimmung von Anforderungen an einrichtungsübergreifende Fehlermeldesysteme (üFMS-B).

[6] Kingston MJ, Evans SM, Smith BJ (2004). Attitudes of doctors and nurses towards incident reporting: a qualitative analysis. Med J Aust.

[7] Kohn LT, Corrigan J, Donaldson MS (2000). To err is human: building a safer health system.

[8] Leistikow I, Mulder S, Vesseur J (2017). Learning from incidents in healthcare: the journey, not the arrival, matters. BMJ Qual Saf.

[9] Macrae C (2016). The problem with incident reporting. BMJ Qual Saf.

[10] Mitchell I, Schuster A, Smith K (2016). Patient safety incident reporting: a qualitative study of thoughts and perceptions of experts 15 years after ‘To Err is Human’. BMJ Qual Saf.

[11] Neale G (2005). Are the risks of hospital practice adequately recognised by incident reporting?. BMJ Qual Saf.

[12] Pham JC, Girard T, Pronovost PJ (2013). What to do with healthcare incident reporting systems. J Public Health Res.

[13] Rall M, Martin J, Geldner G (2006). Charakteristika effektiver Incident-Reporting-Systeme zur Erhöhung der Patientensicherheit. Anaesthesiol Intensivmed.

[14] Shojania KG (2008). The frustrating case of incident-reporting systems. BMJ Qual Saf.

[15] Thomas MJ, Schultz TJ, Hannaford N (2011). Mapping the limits of safety reporting systems in health care—what lessons can we actually learn?. Med J Aust.

[16] Vincent C, Carthey J, Macrae C (2017). Safety analysis over time: seven major changes to adverse event investigation. Implement Sci.

[17] World Health Organization (2005). World alliance for patient safety: WHO draft guidelines for adverse event reporting and learning systems: from information to action.

